# A step forward in health-related IoT cybersecurity: remarks on the proposal for a liability for defective products directive

**DOI:** 10.3389/fdgth.2023.1193255

**Published:** 2023-10-04

**Authors:** Jarosław Greser

**Affiliations:** Faculty of Administration and Social Sciences, Warsaw University of Technology, Warsaw, Poland

**Keywords:** medical device, H-IoT, product liability, cybersecurity, mental health, mental health regulations, EU law, internet of things

## Abstract

This article outlines the efforts of the European Union in health-related IoT (H-IoT) cybersecurity. The first part identifies cyber threats that are specific to H-IoT. The second part covers the overall regulatory picture and briefly addresses both existing law and legislative initiatives. The third part discusses the Proposal for a Liability for Defective Products Directive and the measures it contains that relate directly to H-IoT.

## 1. Introduction

Health-related IoT (H-IoT) covers a wide range of clinical, consumer, and research applications. Such solutions are used frequently in healthcare to support practitioners and patients, both in emergency cases and in the treatment of chronic illness (1). They also contribute to reducing healthcare costs and improving the standard of care for the chronically ill (2). Research demonstrates that such solutions can also be used in the treatment of mental illnesses. Positive results have been reported in the diagnosis and treatment of bipolar disorders, depressive disorders, schizophrenia spectrum disorder, and stress-related disorders (3). H-IoT is also used in the treatment of Parkinson's disease and other neurodegenerative diseases (4) It can be assumed that the number of H-IoT applications will grow in view of the confluence of factors that have led to a rapid increase in IoT devices. It is estimated that by the end of 2027, IoT reached 29 billion connections (5). Global investment in the deployment of the technology fluctuated around USD 740 billion in 2020, with prospective growth of several dozen percent in the following years (6). By 2030, around 60% of IoT solutions will be used for or by consumers, including a significant proportion in healthcare (7).

A plethora of ethical, legal, and cybersecurity risks are associated with the use of H-IoT. This article addresses cybersecurity by demonstrating how threats can be mitigated through regulatory requirements. The article comprises three parts. The first discusses the causes and characteristics of H-IoT threats. The second presents the broad legal context that governs H-IoT cybersecurity in the European Union (EU). The third analyzes the Proposal for a Liability for Defective Products Directive adopted by the EU Commission in September 2022, which is intended to improve H-IoT cybersecurity.

## 2. Characteristics of cyber threats to H-IoT

When analyzing the threats that surround H-IoT, it is important to note that in healthcare, patients use a combination of consumer health and fitness IoT devices, and prescribed medical IoT devices (8). Although their degree of security varies due to the different regulatory requirements in the marketing of specific products, some vulnerabilities are shared by all solutions (9). These includes limitations that result from devices’ design, the policies of manufacturers, and the risks specific to the environment in which they operate.

The most significant limitation related to many IoT devices is that they are battery powered. This results in severe constraints on power, memory, and processing resources (10). In practice, this limitation impedes the use of resource-consuming programs, such as standard operating systems, firewalls, and antivirus software that significantly increase the degree of security (11). Another issue that affects IoT that is embedded in other products is the difficulty in accessing it (12). This prevents users from installing software patches, or even from rebooting their devices.

The market for IoT products is highly competitive. This forces reductions in the time spent on research and testing (13). Simultaneously, using hardware and software components that were designed for other purposes is a common practice (14). This can create security gaps. Large numbers of devices with the same vulnerabilities can increase the impact of successful attacks (15). A separate problem is so-called “orphan devices”: solutions that remain in use despite the absence of support from manufacturers—in particular, the release of security upgrades (16, 17).

Irrespective of other factors, H-IoT devices are vulnerable in areas specific to the medical sector. According to an FBI report, health services are the most common target of ransomware attacks in 2021 (18). The digitalization of healthcare associated with the COVID-19 pandemic has had a significant impact on cybercrime, with data showing a 600% increase in activity during the pandemic (19).

H-IoT technology can serve both as a target and as a vector for attacks. In the former case, this could involve the theft of data from devices or the intentional disruption of their functioning, which, in extreme cases, could result in the deaths of patients^1^. In the latter case, vulnerabilities in devices can offer a route to accessing the internal networks of healthcare providers, and, thus, allowing access to confidential resources (22) or being used to launch DDoS attacks (23).

## 3. The legal context that governs H-IoT cybersecurity in the European Union

The EU framework comprises several pieces of horizontal and sectoral legislation that cover aspects linked to cybersecurity from different angles: products, services, and crisis management (24). These include data protection and data governance regulations, among which GDPR is a leading one. Horizontal regulations are based on the 2016/1148 Directive (NIS Directive) and the recently adopted 2022/2555 Directive (NIS 2 Directive). The former focuses on building an environment that supports cybersecurity rather than imposing direct obligations on H-IoT manufacturers (25). The latter, which should be transposed by EU Member States by October 2024, provides for a broader scope of application, but is generally limited to H-IoT devices that are classified as medical devices. For the (CER Directive) (26) also applies to medical device manufacturers, but only those considered critical within the meaning of Article 22 of Regulation EU 2022/123 (27).

Among the sector regulations for H-IoT, there is a clear division between products that are classified as medical devices and other solutions. The former are governed by Regulations 2017/745(MDR) (28) and 2017/746(IVDR) (29). The term “cybersecurity” is not referred to directly in either of these acts. We can interpret the obligations in this area from the general principle that requires product security: Article 5 (1) of the MDR and Article 5 (1) of the IVDR and the regulation of IT systems used in medical devices i.e., art. 17.2, 17.4, 18.8 of Annex 1 MDR (25 p. 9). Both acts also contain detailed rules on post-market surveillance, which includes the obligation to create post-market surveillance plans, periodic safety update reports, analyses and reporting of serious incidents, trend reporting, and analysis of vigilance data. According to the Medical Device Coordination Group's guidelines, post-market surveillance also covers cyber threats and incidents caused by cyberattacks (25 pp. 28–30). Thus, it can be considered that medical device regulations address cybersecurity issues although it is debatable whether these issues should be addressed directly and whether the requirements are sufficient. Nevertheless, it should be underlined that they constitute a comprehensive regulation that must be applied to H-IoT devices that are classified as medical devices. It must be noted that manufacturers are no obligation to have any product certified as a medical device—even if the purposes of its operation relate closely to medical uses, which include the diagnosis, prevention, monitoring, prediction, treatment, or alleviation of disease, injury, or disability. Although this may exclude such a device from use by health professionals, it does not limit its availability in the consumer market.

For H-IoT devices not classified as medical devices, the determination of cybersecurity requirements is even more complex. Presently, there is no legal act that comprehensively regulates this issue. The European Commission has recently proposed a solution to address this matter: the Cyber Resilience Act (CRA) (30). The legislative process is pending and there is no certainty that it will be completed within the current term of the European Parliament. A similar caveat should be attached to the proposal for the General Product Safety Regulation (31), which introduces new rules on product security for products that are not covered by other legislation. One motive for legislative changes in this area is the widespread application of IoT, which is insufficiently covered by current product safety regulations.

Among the legal acts currently in force, it is necessary to highlight Commission Delegated Regulation 2022/30 (32), which will apply from August 1, 2024. This is an implementing act to Directive 2014/53 (33), which regulates radio devices. It applies to internet-connected radio equipment, understood as any radio equipment that can communicate over the internet, whether it communicates directly or via any other equipment. Thus, the regulation will be applicable to the vast majority of H-IoT devices, but not to medical devices regulated by MDR and IVDR, which are explicitly excluded from the scope in Article 2. The regulation requires manufacturers to design products in such a way that the devices neither harm networks or their functioning nor misuse networks’ resources. Devices that process personal or location data are required to have built-in safeguards to protect their users and subscribers. The same requirement applies to internet-connected radio equipment, wearables, and child surveillance systems, regardless of whether they process personal data. Devices that are launched on the market before August 1, 2024 can be sold and will not need to be modified to comply with the new requirements.

The degree of H-IoT cybersecurity is affected directly by the availability of software updates. Changes in this field were introduced by Directive 2019/771 (34), which is part of European consumer law. It applies to “goods with digital elements”, which includes H-IoT devices. According to Article 7 (3), the seller shall ensure that the consumer is informed of and supplied with updates, including security updates, that are necessary to keep those goods in conformity, for the period of time that the consumer may reasonably expect. Recital 31 states that the period is to be assessed based on the type and intended use of the goods, but is generally not shorter than the producer's product liability, and may be longer depending on the circumstances. This arrangement is intended to limit the operation of orphan devices, but does not eliminate them. In addition, the duty to install updates falls on the user, which may not be possible or may entail significant difficulty for some H-IoT devices. The user also has the right not to install updates with the result that, according to Article 8 (3), the manufacturer is released from liability. Such a regulation may give rise to highly undesirable consequences in H-IoT security. It finds its justification particularly in situations in which an update contains, in addition to security fixes, other changes that the consumer does not want to agree to—for example, the changing of the interface, the addition or limiting of functionality, or reductions in the degree of privacy. Practice shows that updates are usually delivered in packages without detailed descriptions of the changes, and have an “accept or reject” character. However, in the case of patches that relate solely to the security of a device, the possibility of rejecting the patches can threaten both the user and the network.

## 4. Proposal for a liability for defective products directive

Establishing legal requirements for H-IoT cybersecurity is a cornerstone of user protection. To be effective, it is necessary that a system of redress for damages resulting from device's non-compliance be established. Tort or contractual liability provisions, which are regulated in the legislation of EU Member States, may be used for this purpose. The European Commission considered such tools inadequate to guarantee sufficient protection for those who have suffered health or property damage caused by defective products. This was behind the adoption of the Product Liability Directive (35) in 1985, which introduced uniform measures of protection among EU Member States. The legislation was evaluated in 2018 as part of the Commission's Regulatory Fitness and Efficiency Program (36), which showed that the legislation was generally an effective instrument, but that some of the concepts used were inadequate for products in the modern digital economy—particularly those that need software or digital services to function. Moreover, problems that had arisen from the burden of proof for those injured by smart products and the limitation of the claim for property damage to situations in which it exceeds 500 euros were highlighted. In effect, on September 28, 2022, the Commission tabled a proposal for a new directive on liability for defective products (37).

In Article 1, the directive establishes common rules for the liability of economic operators for damage to natural persons caused by defective products. It should be noted that the concept of a “natural person” is broader than that of a “consumer”, thereby guaranteeing better protection for injured parties without having to establish their status with the producer or seller. Recital 17 clarifies that the concept of personal injury includes medically confirmed harm to psychological health, which is relevant from the perspective of H-IoT. The proposal does not limit the size of the claims that can be sought. These changes place H-IoT users in a much better litigation position and allow them to claim the full spectrum of damages that can be caused by such devices.

The compensation option applies only if the damage was caused by a defective product. As defined in Article 6 of the proposal, a product is considered defective when it fails to provide the safety that the public at large is entitled to expect. The provision contains an enumerative list of circumstances that automatically classify a product as failing to meeting this condition. From the perspective of cybersecurity, the condition specified in Article 6 (1) (f), which refers to safety-relevant cybersecurity requirements, is particularly important. This provision should be interpreted in such a way that products should meet all relevant legal obligations. This expectation also appears to include products’ compliance with recognized industry practices, public authority guidelines, and codes of conduct. Such documents are not legally binding in principle, but nevertheless create a standard of cybersecurity practices; compliance with them can reasonably be expected by users.

The proposal introduces a presumption of product defectiveness in certain situations. One of these is a claimant's establishment that a product fails to comply with mandatory safety requirements under EU or national laws that are designed to protect against the risk of damage. In such cases, it will not be possible to refer to noncompliance with nonlegal standards as a source of damage. This seems to be the wrong approach, considering the dynamics of changes in cybersecurity threats and, generally speaking, the more rapid adaptation of nonlegal standards to them. This does not prevent plaintiffs from pleading noncompliance with the standards, but the inability to benefit from the presumption may place defendants, which will often be large firms, in a more advantageous litigation position.

Exemptions from liability have been provided for operators in situations in which a defective product causes damage. One of them is the probability that the defect did not exist when the product was placed on the market. Article 10 (2) (c) indicates that this does not apply if a product's defectiveness is due to the avoidance of software updates or upgrades necessary to maintain safety. Such a provision is very positive from an H-IoT security perspective. It is also a clear indication of the legislator's intent, which resolves liability issues for orphan devices at least during the period in which the consumer could reasonably expect such support. Nevertheless, some issues remain unresolved and may raise interpretative doubts. These include cases in which a defect arose as a result of the use of a “zero-day exploit” that was unknown to the manufacturer or the period in which the update should be released. Despite this, the solution should be considered a step in the right direction; one that increases the degree of protection for H-IoT users.

## 5. Conclusions

Cyber threats to H-IoT devices are a genuine challenge for all stakeholders. The cybersecurity issue on the EU's agenda encompasses such devices. Appropriate regulations can increase their security and lead to better protection of the interests of all parties. Moreover, they can affect all of the causes that lead to the relatively low security of IoT—in particular, the changing of producers’ policies.

The base problem with H-IoT devices is their legal status. It is possible that two products with the same functionality will be subject to different legal obligations when one of them is registered as a medical device and the other is not. In the case of medical devices, the requirements for manufacturers are relatively clear and derive from the MDR and IVDR. This does not mean that interpretive uncertainties do not exist, but it can be assumed that there is a backbone of cybersecurity requirements for H-IoT manufacturers. It applies both to products’ release onto the market and operation throughout their lifecycles. These regulations are reinforced by the obligations imposed on manufacturers of medical devices and healthcare providers, such as those found in the NIS 2 Directive or in the proposal for CER Directive. For consumer products, the situation is less favorable. This is due to the absence of relevant regulations, which are either at the proposal stage (like the Cyber Resilience Act or General Product Safety Regulation), or have been adopted, but will come into force at a later date (like Commission Delegated Regulation 2022/30). Nevertheless, a trend towards comprehensive regulation of the legal situation of IoT is observable. The Proposal for a Liability for Defective Products Directive, which should be appreciated, also forms part of this. However, it is important to note that the directive lies at the beginning of the legislative process and its final form may differ significantly from the proposal.

In view of the challenges posed by consumer H-IoT, legislative work concerning this matter should be given higher priority so that it can be completed during this term of the European Parliament. Otherwise, the period for its entry into force may be significantly prolonged, which may have a negative impact on the level of cybersecurity. It should also be noted that there is no obligation to certify a device as a medical device, even if it can clearly be used for such purposes. The current provisions protecting users appear to be insufficient in view of the development of the H-IoT market. Therefore, an evaluation of the relevant rules is needed as soon as possible.

## Footnotes

^1^There are reports of deaths caused by a cyberattack, but they do not involve a situation involving a direct attack on an IoT device (20). However such a possibility has been proven experimentally (21). It is also possible that such cases have occurred, but have not been reported or have been incorrectly categorized.
